# Supplementary material for: “I wish they heard my story rather than my conditions.” –A qualitative exploration of young people’s experiences during mental health assessment in the UK
Source: PLOS Ment Health. 2025 Sep 18;2(9):e0000436. doi: 10.1371/journal.pmen.0000436 (PMC12798412; doi:10.1371/journal.pmen.0000436)
Supplement: S1 File — (DOCX) [file pmen.0000436.s001.docx]

**Interview Topic Guide**

Before recording starts:

Introduction: “Hi my name is [NAME], and I am a MSc student at UCL. This interview is part of my dissertation research exploring the experiences of young people who have been assessed by mental health services. For the next hour or so, we will be having a chat that is guided by some broad questions, which will help me understand your experience. You are very welcome to add your own thoughts and reflections at any point and will also get a chance to add things that I might have missed at the very end.

- Before we can start the interview, we should go over the consent form. I will be reading out the individual segments to you and you may indicate to me whether you agree or not.
  - Are there any questions regarding the consent form or information sheet?
  - Obtain inform consent.
- The results of this interview will be used to inform my analysis of how young adults have experienced mental health assessments and what those meant to them. They will be published in a peer-reviewed journal and hopefully inform/inspire future research on this topic. There is not much research on this topic yet, so these interviews primarily explore and make way for future research.
- “There are no right or wrong answers. This is solely about how you experienced assessment. You may be as critical or positive as you like.
- “You are *not* obligated to talk about why or for what mental health issue you were at the assessment for. Please only disclose what you are comfortable with. The focus of this project is to get a general understanding of your assessment experience, irrespective of the specifics of your mental health.”
- “If anything does not make any sense to you, feel free to ask for clarification. You may also skip any question you do not want to answer.”
- “Please try not to disclose any personal details of yourself or the person that might have assessed or referred you.”
- Take Name + Age
- “Are there any questions at this point?”

Interview begins:

On question design: *This piece of work is broadly exploratory, thus, there are less preconceived notions and expectations about expected themes. However, some of my experiences as a practitioner have informed points of interest, for example, wanting to understand their expectations around assessment (i.e., diagnosis), the sense-making of assessment and potential aversion to segments of the assessment.* *Additionally, some ideas were theory-driven, in so far as to ask to about/allude to barriers and facilitators to assessment and assessment/first treatment attendance.*

General prompt used throughout the interview:

- Could you expand on that/tell me more about that?

**PART 1**

**Pre-Assessment Experience & Referral**

“How were you referred to your mental health assessment?”

- Follow-up: If referred by other, how did that feel/did you feel that was appropriate?
- Follow-up: If self, what prompted you to refer?
  - Follow-up: Were there any challenges to you being referred?
  - Follow-up: Did you receive any support when being referred?

“Before you attended your assessment, what were your initial expectations?”

- Follow-up: Why do you think [EXPECTATION] would happen?
- If diagnosis was mentioned as an expectation: *Why was that important to you?*

“What did it mean to you to have an assessment appointment within a mental health service?”

“At any point before the assessment, did you have any doubts about attending?”

- Follow up: if so/not, why is that?
- Follow up: If so, how did you overcome these doubts?

**PART 2**

**Experience During the Assessment**

“How did your assessment take place?”

- If unclear: “Was it done remote or face-to-face?”
- If remote: Was it a telephone call or a video call?
  - Follow up: Did the assessment being remote/face-to-face affect you at all?

“Do you remember how your assessment was structured, did it have any noticeable parts?

- Could give examples: “Did you have different segments, such as a part for questionnaires, risk assessment or treatment plans?”
  - Follow-up: How did you feel about the questionnaires and the way they were presented?
- Follow-up: Did that structure influence your ability to share your experience at all and how so?

“What was important to you to get across during the assessment?”

- Follow-up: Why was [THING] important to you?
- Were you successful in getting that across?
  - If yes: Did anything help you with that?
  - If no: Did anything in particular make it difficult for you?

“During the Assessment, did you feel that your experience was heard and understood?”

- If no: What do you wish were different/Was anything missing?
- If yes: what made you feel understood?

“Was there any part of the assessment that you disliked?”

“Was there any part of the assessment that you particularly liked?”

Going into treatment, has your experience during assessment impacted your experience/expectations of treatment at all?

**PART 3**

**Post-Assessment Experience & Reflection**

“How did you feel after the assessment was over?”

- Follow-up: Did you feel like it lived up to your expectations?
  - Why (not)?
- Follow-up: Did you understand what the next steps were to receive help?
  - If next steps included treatment: Did you feel the assessment prepared, you sufficiently/set up the right expectations?

“Overall, with your knowledge of your assessment now and with everything we have discussed today, do you have any reflections about your assessment?”

PART 4

End of Interview

“Is there anything you wish to share about your experience that you think might be relevant but has not been said yet?”

“Do you have any questions for me?”

“Thank you so much for your time. You will be receiving an email with support resources from me; in case you require further mental health support.

[If participant indicated they wish to receive copy of result during consent segment]: Once the findings have been written up, we will be sending you a summary of the results. Your email address will be deleted from our database once we have sent the summary.

I will stop recording now.”

Stop recording.

Give debrief information, if necessary.

End interview.
