# Supplementary material for: “I wish they heard my story rather than my conditions.” –A qualitative exploration of young people’s experiences during mental health assessment in the UK
Source: PLOS Ment Health. 2025 Sep 18;2(9):e0000436. doi: 10.1371/journal.pmen.0000436 (PMC12798412; doi:10.1371/journal.pmen.0000436)
Supplement: Attachment — Submitted filename: Response to Reviewers.docx [file pmen.0000436.s003.docx]

Reviewer(s)' Comments to Author:

| Editor | Author response |
| --- | --- |
| 2. There are some concepts in this paper that need revising and greater clarity e.g. the conclusions suggest the idea that the medical model is separate to person-centred care – I suggest that the authors do more reading and across the paper clarify the definitions and critiques.  &  11. There are some concepts in this paper that need revising and greater clarity e.g. the conclusions suggest the idea that the medical model is separate to person-centred care – I suggest that the authors do more reading and across the paper clarify the definitions and critiques. | Thank you for the insightful comment and the provision of further resources. After careful consideration of the literature, we have made some amendments to the discussion section to mention Lines et al’s (2015) differentiation of care models and the ways in which participants may have resonated with person-directed care, as opposed to person-centred care:  *“Crucially, participants felt that had they been given more agency by having direct control over aspects of the assessment; for instance, being able to choose the assessment modality or pick a consequent treatment, they would have been more engaged. Increasing people’s sense of control during assessment by adopting a collaborative approach that facilitates shared decision making may thus engage people more and subsequently reduce attrition (61, 62). This sentiment resonates with Lines et al.’s (59) idea of person-directed care. An advancement of person-centred care, person-directed care not only respects the person as a holistic entity beyond the medical context but actively encourages the person to take control of and make meaningful decisions about their care plan, empowering the person to be part of their recovery.”* p32  Upon further reflection and considering the depth and nuance required to discuss this issue responsibly, we have decided to remove the mention of the medical model from the manuscript altogether as it has only explicitly appeared once at the tail end of the paper and was not paramount to the central claims of the research. We do agree that this may warrant a larger investigation in the future, but other comments have pointed to the excessive use of citations and length of this paper, which would be further exacerbated by the addition of this discussion. |
| 3. Please revise the whole manuscript carefully to ensure there are no grammatical errors and paying attention to the language styles used – there are several examples of use of informal English – e.g. p 6 Gotten in touch should be rephrased more formally | Thank you for this, we have since amended the grammar and language style of the paper to conform to the journal’s academic standards. |
| 4. P30 “for a lot of participants” – This is poor grammar – for many participants. | Thank you for this suggestion. This has been changed. Similar changes were made on P15 & P29 where the same syntax was encountered. |
| 5. There are also some examples of poor grammar e.g. p 30 his misalignment of clinicians’ goals and patient goals creates and ostensible power imbalance that that makes self-disclosure harder | Thank you for pointing this out. The sentence has been updated as follows:  *“This misalignment of clinicians’ and patients’ goals creates an ostensible power imbalance that makes self-disclosure harder.”* |
| 6. The manuscript is longer than it need to be with some sections using many more words than required to convey meaning. E.g. Strengths and limitations section could be expressed much more compactly. | We agree that some parts are indeed long, especially in the strength and limitations sections. We have rewritten large sections of the manuscript to make them more succinct and remove jargon. |
| 7. The references total over 70 papers – please edit the paper and citations to a maximum of 50 citations that are most pertinent to support your points | We appreciate concerns around concision and have aimed to make the text more succinct. References are used to further support statements, and double or triple references have been used where deemed appropriate to substantiate important claims. Most of these citations are pertinent in grounding the current work in the contemporary scientific field, lending credence to their veracity. We do believe that the chosen citations add to the scientific rigour of the paper and allow interested readers to further investigate the claims made.  Furthermore, the PLOS Mental Health guidelines do not appear to limit manuscripts to 50 references. The journal has also recently published articles with a higher number of references e.g.:   1. Low G, França AB, Gao Z, Gutman G, von Humboldt S, Allana H, Wilson DM, Naz A. Anxiety symptoms and coping strategies used by older adults during COVID-19: A national e-study of linkages among and between them. PLOS Mental Health. 2025 Apr 29;2(4):e0000304.   We do, however, agree that some citations may have been superfluous and have reduced the overall citation count from 71 to 60 (including two new references as per reviewers’ requests). |
| 8. Was there any effort to ascertain whether participants were from disadvantaged socioeconomic groups? | Unfortunately, we did not ascertain whether participants were from a disadvantaged socioeconomic status. We have added a sentence to the limitation section to reflect the importance of SES:  *“Nonetheless, this project did not specifically recruit people from a disadvantaged socioeconomic background, which may further limit assessment access and beget negative experiences.”* p34 |
| 9. P35 Please review conclusions to make more moderate claims e.g. ‘assessment are plagued’ could perhaps be stated ‘ are limited by … “ a. Eg. , new assessment care practices need to be conceived that prioritise person-centred care, synthesising both it and the medical model into one  b. Could be said “new assessment care practices should prioritise person-centred care” | We agree that some of the conclusions should be more tempered. The suggested sentences have been changed to:  *“Currently, assessments are perceived as largely depersonalised hurdles that are limited by systematic communication barriers and an absence of agency”* p36  &  *“To fulfil that potential and meet people’s demands, new assessment care practices should prioritise person-centred, or even person-directed care, where possible.” p36* |
| 10. I appreciated the thoughtfulness and reflexivity in the positionality statements offered, although I wondered whether reflexivity around researcher ethnicity and other markers of social hierarchy could also be reflected on. | Thank you for this suggestion. We agree that reflexivity is an integral part of qualitative work and have since expanded the reflexivity section to make further note of the researcher's ethnicity and its implications for the research.  Aside from making note of the researcher's ethnicity and queer community status, we also included the following paragraph:  *“AD’s educated and ethnically privileged background may have introduced power imbalances during the interview, which could have discouraged participants from sharing culturally sensitive experiences. Furthermore, as a white man, AD may not be privy to the nuances of marginalisation, which may have been absent from the topic guide or follow-up questions during the interviews. “* |
| Reviewer 1 |  |
| 1. Revise the sentence structures and rephrase certain words in paragraphs, avoid Jargon. | Thank you for this comment. We agree that some paragraphs should be phrased differently and have adjusted the text by removing jargon and checking for grammar**.** |
| 2. Also add one or two objective analysis/summary of results to make more clearer senses of the statistics in Table 1. Breakdown of demographic data of study participants. For example what does this mean in terms of over all mental health in the demographic regions, why has these figures impacted mental health in self assessment in general? Why are percentages lower in certain demographics than others? Write a small analysis underneath to add to the research.  &  Expand on the results with a summary to make it clear for readers to digest and understand the figures in the table to be precise and concise,. | Thank you for these comments. Although intriguing, and certainly worthy of further investigation within the contexts of a different methodological approach, the current study is qualitative in nature. Demographic statistics were primarily provided to aid prior claims about recruiting a diverse sample. The current amount and type of data does, hence, not lend itself to quantitative analysis and any inferences (i.e., relations of these figures to mental health impacts) would be inconclusive/no advisable. Further analysis of the demographics within this small qualitative sample would thus be spurious. |
| 3. Double check spelling, commas, headings are correctly formatted with the paragraphs especially with the personal quotes of participants, remove the lines for example. | Thank you for this suggestion. We have since double checked spelling, commas and headings to be correctly formatted.  As for the lines in front of the indentations made for quotes, this is standard practice for most qualitative research articles and serves to further distinguish the quotes (participant data) from the interpretative body of the text. This is consistent with recently published papers in this journal, for example:   1. Eager, S., Lloyd-Evans, B., Bousfield, J., Downey, M., Harrison, I., Killaspy, H., & Mezey, G. (2025). Barriers and facilitators to social inclusion among people with severe mental illness: A qualitative study. *PLOS Mental Health*, *2*(4), e0000137. |
| 4. Double make sure references are cited properly and are in correct alphabetical order with links to research for works. (Accessed at 12/03/2024) for example. | Thank you for this comment – we have double checked that all the references are formatted in accordance with journal guidelines. They do not need to be in alphabetical order, nor do they require a “last accessed” date as this journal requires Vancouver referencing style. |
| Reviewer 2 |  |
| 1. However, author should clarify how coding disagreements were resolved, when multiple researchers code the same data, differences in interpretation are common. So, Inter-Coder Reliability Score is recommended. | Thank you for this suggestion. Although it is true that some forms of thematic analysis employ a kappa reliability score to denote agreement in coding, reflexive thematic analysis does not (Braun et al., 2023). More specifically, this form of thematic analysis uses a second coder to further increase the breadth of themes available and ensure that more than one perspective has been considered during the analysis process. Whether or not coders agree on topics is secondary to the main goal of considering perspectives outside of one’s own remit. |
| Reviewer 3 |  |
| 1. The current definition of "mental health assessment" is overly broad and would benefit from a more detailed description. In practice, mental assessments are conducted at varying levels of specialization by providers with different expertise. For example, an interview conducted by a psychiatrist may evoke different experiences and emotional responses compared to a questionnaire-based assessment administered by a social worker or less specialized care provider. | Thank you for this comment. It is true that the provided definition of mental health assessments is broad. This has been done by choice. To our knowledge, there is no unilateral or specific mental health assessment definition within the literature that encompasses the entirety of mental health assessment, which, in part, inspired this research. They are largely underdetermined and non-standardised. We appreciate that this has not been made sufficiently clear, thus, we have added the following sentence:  *“Across domains, mental health assessment appears to be largely underdetermined and non-standardised, whereby different service providers, i.e., primary or secondary care may follow different, service specific protocols.”* p4  In addition, just as you had mentioned, depending on the clinical context (i.e., psychiatrist nurse etc.), assessments may differ substantially. We had noted the lack of differentiation as part of our limitations and have amended the following sentence:  “However, the UK’s mental health service landscape is varied and nuanced, including psychiatric assessments, Talking Therapies assessments, mental health nurses’ assessment or GP assessments. All of these may differ in nuanced and meaningful ways, and these differences may have not been captured in this study.” p34 & 35  However, the purpose of the current study was largely exploratory, which is how we justified this lack of differentiation. |
| 2. The identified themes seem to imply that the focus of the investigation was primarily on assessments using psychometric tools. If that is indeed the case, the authors should clarify and explicitly specify the types of mental assessments being discussed to better capture these distinctions. | Thank you for this comment and suggestion. Although issues with psychometric tools were a prominent theme across participants, they were not the exclusive focus of assessments. In general, participant experienced assessment that were a combination of psychometrics and psychosocial conversation. For example, participants have voiced strong attitudes towards being listened to actively and treated with warmth, as is reflected in the “Safe Space” theme. To ensure participant anonymity, we did not elicit any information regarding the specific type of assessment they had attended. |
| 3. The level at which disengagement occurs is also crucial and warrants discussion. It is important to differentiate whether assessments are conducted for checking eligibility for a specific service, during emergency triage by a nurse, or within a consultant or psychiatrist’s clinic. Additionally, clarification is needed on whether disengagement happens at the first encounter with mental health services or as a result of multi-step, specialized assessments. . | Thank you for this insight. We agree that it would be beneficial to further investigate the type and stage of assessment to understand the nuances of assessment and disengagement better. For the exploratory purposes of this study, we determined to not elicit this information. In addition, the purpose of assessment, i.e., checking eligibility, building rapport etc. is largely undefined and underdetermined. In our limitation section, we have aimed to mention the lack of focus on a specific assessment type and have amended the following sentence to add further clarification:  “However, the UK’s mental health service landscape is varied and nuanced, including psychiatric assessments, Talking Therapies assessments, mental health nurses’ assessment or GP assessments. All of these may differ in nuanced and meaningful ways, and these differences may have not been captured in this study.” p34 & 35 |
| 4. Furthermore, the level of training that assessors have in administering psychological assessments is a significant factor that could influence the experience of agency and subsequent disengagement. If these aspects were not captured in the data collection, they should at least be discussed as limitations | Although interesting and certainly relevant to the assessment that participants received, we did not collect any data on the training that the assessor received as this study was focused on understanding the experiences of those who have received assessments, rather than those who administer them. Since participants may not have known the extent to which the assessor may have been trained, eliciting this data from participants may result in unreliable outputs. However, it could have been interesting to further investigate the type of professional that administered the assessment in a follow-up study. |
| 5. Since the study was conducted in the UK, it would be beneficial to provide a brief overview of current practices in mental health care and assessment within the country. Describing the typical pathways (e.g., referrals from primary care, the structure of emergency versus routine assessments in public versus private sectors, and how assessments are integrated into overall care) would help contextualize the findings and clarify whether they can be extrapolated to other settings. Noting any policy or systemic factors unique to the UK could further illuminate the specific challenges faced by young people in this context. | Thanks for this suggestion. We agree that some context to the mental health service setting may be useful, however, for the sake of concision, we decided not to add extensive context to the manuscript. Since, as you have pointed out, UK health service settings are quite varied, it would be difficult to describe all of them in detail, especially because this study did not target any one context. Instead, we added a short sentence to the introduction:  *“Across domains, mental health assessment appears to be largely underdetermined and non-standardised, whereby different service providers, i.e., primary or secondary care may follow different, service specific protocols.”* P4  In addition, we have also amended the following sentence in our limitations section to further reflect the breadth of service contexts and our lack of specification:  (Sentence in comment 3) |
| 6. The theoretical framework of the study should be specified in the Methods section. It is important to clarify whether any predetermined theories or conceptual frameworks were employed to contextualize the phenomenon under study and to guide the inductive analysis. | Thank you for the comment. We agree that transparency regarding our epistemic and methodological assumptions is important. The current study was exploratory and did thus not employ any pre-determined constructs or theories. As is common for inductive research, it is primarily data-driven, as opposed to hypothesis driven. This is reflected in the exploratory label as well as the choice of conducting reflexive thematic analysis, as opposed to codebook-analysis, for example. Nonetheless, another sentence was added to make this theoretical agnosticism salient:  *“As such, no predetermined theories or themes were applied to the data and analysis was inductive.”* p8 |
| 7. The positionality of the researcher should be more thoroughly described. Given that factors such as race/ethnicity and sexual orientation are relevant to the research, it would be beneficial to clarify how the researchers and interviewers are positioned in relation to these diverse backgrounds. | Copied response to editor comment 10:  Thank you for this suggestion. We agree that reflexivity is an integral part of qualitative work and have since expanded the reflexivity section to make further note of the researcher's ethnicity and its implications for the research.  Aside from making note of the researcher's ethnicity and queer community status, we also included the following paragraph:  *“AD’s educated and ethnically privileged background may have introduced power imbalances during the interview, which could have discouraged participants from sharing culturally sensitive experiences. Furthermore, as a white man, AD may not be privy to the nuances of marginalisation, which may have been absent from the topic guide or follow-up question during the interviews.”* P10&11 |
| 8. There appears to be some confusion between the terms "gender" and "sex." Sex generally refers to biological and chromosomal differences, typically classified as male or female, whereas gender is a social, personal, and cultural construct that pertains to an individual’s self-identified identity and may not conform strictly to traditional labels. It is particularly important to use appropriate lexicon and clarify this distinction. I recommend that the authors clearly define both terms and use them consistently throughout the manuscript to avoid conflation. | Thank you for pointing to the importance of the distinction between sex and gender. We aimed to only refer to participants' gender and the self-identified labels that have been provided by the participants themselves. However, we do acknowledge that this conflation does exist in the demographics table, which has now been adjusted to refer to the labels “man” and “woman”, as opposed to “male” and “female”. |
| 9. In the second paragraph, the manuscript briefly mentions that young people may be hesitant to disclose sensitive information due to stigma or mistrust. I suggest expanding on this point by providing additional context, such as contrasting the experiences of stigma and mistrust faced by young people with those of older age groups, and referencing relevant literature. | Thank you for this comment. Although interesting, contrasting youth experiences with experiences of older populations does not add to the argument in the introduction that youth face these issues. We do not believe that their stigma is further validated by contrasting it with other people’s stigma. Since other comments have already pointed out the extensive length of the manuscript, we decided to not add further details on this point. |
| 10. The introduction describes “young people” as those aged 10–24, yet the methods specify a sample of young adults between 18 and 26. This inconsistency in age definitions should be addressed. I recommend clarifying the age range according to the study’s objective and ensuring consistent terminology across all sections. | Thank you for this accurate observation. It is true that, in the literature, young people are defined as those aged between 10-24. However, due to the resources and time available for the current project, only a range of those could be recruited from for this study.  To provide further clarity, we have added a sentence in the limitations section of the manuscript:  *“Moreover, even though this project’s remit is defined within the context of young people, due to feasibility issues, no people below the age of 18 were recruited. In the future, it would thus be important to hear from younger people as well.” p*35 |
| 11. It is advisable to add a reference for choosing the specific age bracket (18–26) for young adults. | We agree that a citation was missing. We have added the following reference to support our choosing of the present age bracket:  Breiner, H., Stroud, C., & Bonnie, R. J. (Eds.). (2015). Investing in the health and well-being of young adults. |
| 12. The final paragraph in the "Lack of Agency" subsection introduces an important discussion about differences in the perceived sense of agency between private and public services; however, this argument would benefit from further elaboration and stronger support with direct participant quotes. | Thank you for this suggestion. We agree that this is an interesting remark and deserves further scrutiny. As such, we have added a full participant quote to this claim and have added a rewritten paragraph to follow along:  Participants who have had experiences with both public and private mental health services also suggested that agency is a financial feature exclusive to private care.    “Yeah, I think because ultimately, when you go privately, you're paying for a service, so then you have more of a stake and sense of being able to say if the service is adequate or not...And I guess, yeah, you have the power to choose, to some extent, because of money, etcetera, who you go to and what you want to share...whereas obviously in the NHS you just get put with whoever, wherever.” (YP004, non-binary)”    Financial investment is perceived to bestow participants with the power to choose their care pathways and the types of information they disclose. By contributing financially, participants feel as though they have paid for the ability to direct their care, as they are holding a stake in the service. Therefore, agency may be seen as a feature absent in public care. The absence of financial investment may further exacerbate perceived power dichotomies and service disengagement. |
| 13. In the “Diversity” subsection, in the first paragraph's first line, the term “sexual identity” should be replaced with “gender identity.” If the intended reference is to aspects of sexuality, “sexual orientation” would be the appropriate term to use. | Thank you for this observation. Sexual identify has been chosen purposefully to represent a large concept that subsumes both sexual orientation, sexual orientation identities and gender identity. It is broadly thought to encapsulate both self-identified gender and sexual orientation as well as group memberships and unconscious sexual desires. Please see:   1. Shively MG, De Cecco JP. Components of sexual identity. Journal of homosexuality. 1977 Dec 1;3(1):41-8. 2. Dillon FR, Worthington RL, Moradi B. Sexual identity as a universal process. InHandbook of identity theory and research 2011 (pp. 649-670). Springer, New York, NY. |
| 14. In the first paragraph, please insert the missing word "of" between "one" and "the first." | Thanks for pointing this out, “of” has been added. |
| 15. On page 30, line 23, remove the extra "that." | Thank you for spotting this. The repeated “the” has been deleted. |
