# Supplementary material for: “I wish they heard my story rather than my conditions.” –A qualitative exploration of young people’s experiences during mental health assessment in the UK
Source: PLOS Ment Health. 2025 Sep 18;2(9):e0000436. doi: 10.1371/journal.pmen.0000436 (PMC12798412; doi:10.1371/journal.pmen.0000436)
Supplement: Attachment — Submitted filename: Response_to_Reviewers_auresp_2.docx [file pmen.0000436.s004.docx]

Reviewer(s)' Comments to Author:

| Journal | Author response |
| --- | --- |
| 1. In the ethics statement in the Methods, you have specified that verbal consent was obtained. Please provide additional details regarding how this consent was documented and witnessed, and state whether this was approved by the IRB | Thank you for your comment. We can confirm that we received approval for this modality of consent from University College London’s Research Ethics Committee (we did not need to seek IRB approval for this research). We have amended the manuscript to clarify how verbal consent was documented and recorded by the interviewer. We have also confirmed in the manuscript that this procedure was reviewed and approved by the UCL Research Ethics Committee. The updated text in the Methods section now reads:  “Verbal informed consent was obtained from all participants prior to the interview over Microsoft Teams and was recorded separately by the interviewer immediately before the interview began. Audio recordings of consent were securely stored on a password-protected, access-controlled shared drive. This consent procedure, including the use of verbal consent, was reviewed and approved by the UCL Research Ethics Committee.” |
| Reviewer 4 |  |
| 1. Although the manuscript acknowledges different types of assessments (e.g., GP vs. psychiatrist vs. talking therapies), it treats them as one conceptual unit. These assessment modalities may differ significantly in tone, setting, purpose and patient experience. Please clarify if participants were clustered by assessment type, and if not, explain why thematic saturation was still appropriate across modalities. | Thank you for raising this point. In this work, we followed Braun and Clarke’s reflexive thematic analysis approach, which is grounded in a qualitative, interpretive paradigm. As Braun and Clarke (2019) note (https://www.tandfonline.com/doi/full/10.1080/2159676X.2019.1704846), the concept of ‘thematic saturation’ is not consistent with the values and assumptions of reflexive thematic analysis, where meaning is seen as generated through the researcher’s active engagement with the data, regardless of the number of data items. We therefore did not aim to achieve ‘thematic saturation’ in this work, but rather to ensure sufficient data richness and diversity to support a meaningful and well-developed analysis.  Moreover, as acknowledged in our limitation section, different assessment modalities may indeed elicit different experiences. Future studies should look at individual modalities to get a better understanding of experiences specific to those assessments. Since this was an exploratory and largely theoretically agnostic project, we did not cluster by assessment type. The assumption here is that, despite their differences, assessments share commonalities across modalities that elicit thematically replete and independent themes. This can be seen within the data, where the lack of personalised care, for example, is apparent and pronounced across all participants, irrespective of the attended assessment modality. To further clarify this, we have added the following two sentences to different sections of the manuscript:  Participants & Recruitment section:  “As this study was exploratory, participants were not stratified according to assessment modalities.”  At the beginning of the Results section:  *“Since participants were not stratified according to different assessment modalities, themes that explored the specifics of a single assessment modality were not further pursued as this was outside of this project’s remit. The identified themes were recurrent, rich and coherent, directly relating to the central aim of this study. This can be understood as different assessment modalities eliciting similar core experiences that warrant an analysis of assessment experiences as a whole.”* |
| 2. The abstract broadly states the findings (e.g., “lack of agency,” “systematic barriers”) without naming any specific examples to increase engagement and specificity (e.g., about psychometric tools or modality mismatch). | Thank you for this suggestion. We appreciate the importance of specificity in communicating qualitative findings. However, we have opted to retain the current level of detail in the abstract to adhere to journal word limits and maintain consistency with other qualitative studies recently published in PLOS Mental Health, which similarly report theme-level findings without specific sub-examples. This approach also allows us to present a clear and accessible overview of the key findings while reserving detail for the main body of the paper. |
| 3. The discussion focuses on critique and literature alignment but lacks policy implications or practitioner recommendations. For a paper on service design and assessment practice, this limits real-world applicability. Please add a paragraph suggesting practical changes for assessors, service managers or NHS policy e.g., structured training in person-directed communication or embedding choice in modality | Thank you for raising this point. We agree that we want to ensure real-world applicability of our findings, however as this is a single qualitative study, we wished to avoid making concrete recommendations that were not based in evidence. However, we agree that it is useful to suggest potential applications, therefore have added the following sentence to the end of the discussion to add more specific, tentative recommendations:  “Although this research has not differentiated between service modalities and can thus not make service specific recommendations, we can make some overarching suggestions on how to improve mental health assessments for young people. Rather than focusing only on improving training for practitioners, it may be more fruitful to implement systemic, service-wide changes that cascade into practice. Hence, services may wish to allow for additional time in assessments for patients to express themselves, removing the focus from structured assessments and quantification of symptoms. This does not imply that symptom questionnaires need to be made redundant, but they should supplement, and not dictate, personal narratives. Additionally, services could give people meaningful choices about their assessments and treatments. For instance, people could make collaborative decisions about their assessment and treatment modality. This may empower people to feel meaningfully involved in the assessment process.” |
| 4. The core measure of assessment success is participant comfort or disclosure but no triangulation with service effectiveness or outcomes. While appropriate for qualitative work, this framing might be read as equating emotional comfort with assessment quality. Please add a short paragraph acknowledging that this study focused on experiential validity and future work could compare these insights with clinical outcomes or follow-through into treatment. | We appreciate this comment; it is true that we are unable to explore the outcomes of assessment in this research, as we focused primarily on people's experiences of assessment (and not outcomes following treatment). Whilst a poor experience at assessment may impact treatment outcomes, this is not something we are able to conclude from this qualitative study. As suggested, we have added a short paragraph to our limitations section to make this limitation clear to the reader:  “Additionally, this study was exclusively focused on capturing participants’ experiences of assessment, meaning we are unable to draw conclusions about the effectiveness of assessments in allocating people to the “correct” treatment, or for treatment outcomes following support. To ascertain the outcomes of assessment as relating to treatment, future studies should incorporate longitudinal designs, following participants through into treatment and compare assessment experiences with treatment outcomes.” |
| Reviewer 5 |  |
| 1. Section 'PPI Involvement' requires more detail | Thank you for this suggestion. We have added additional details to the PPI section to clarify how they were recruited and to explicitly state their contributions to the project:  “Following Jennings et al. (36) framework for best practice for patient and public involvement in qualitative research, two young people with lived experiences of using mental health services were involved with the creation of the topic guide and the analysis of the data. They were members of an existing lived experience advisory group that had previously collaborated with a member of the research team (RA) on a separate project, and were invited to contribute to this study based on their relevant expertise and established working relationship. Two separate online PPI panels were held. In the first panel, contributors suggested revisions to the topic guide to improve clarity and relevance, particularly around language, tone, and the framing of sensitive topics. In the second panel, they reviewed initial thematic summaries and helped shape the preliminary analytic framework. Their insights informed the final coding structure and interpretation of findings.” |
| 2. Findings: the 'Diversity' section seems to imply that two participants and one other share this view. However, is this theme also shared by the rest of the participants, or just by three? Perhaps more clarity here | Thank you for pointing this out. We agree that it is important for readers to get a general feeling of how well represented themes were within the data. However, we want to remain cautious about quantifying the occurrences of experiences within qualitative research as this does not fit the paradigm of reflexive thematic analysis. As such, we opted to not specify the concrete number of people whose experience fit within the theme. To further clarify, we have adjusted the following sentence:  “Several other ~~P~~participants shared that they would have felt more comfortable disclosing their issues, especially those relating to their cultural realities, if they had been paired with an assessor from a similar cultural background. “ |
| 3. References section whereby nos. 6 and 21 seem incomplete. | Thank you for noticing. We have formatted and completed both references. |
